# The complete chloroplast genome of *Rhododendron delavayi* (Ericaceae)

**DOI:** 10.1080/23802359.2019.1689860

**Published:** 2019-12-09

**Authors:** Jie Liu, Ting Chen, Yubin Zhang, Yuke Li, Jiyi Gong, Yin Yi

**Affiliations:** a Key Laboratory of State Forestry Administration on Biodiversity Conservation in Karst Mountainous Areas of Southwestern China, Guizhou Normal University, Guiyang, PR China;; b School of Life Sciences, Guizhou Normal University, Guiyang, PR China

**Keywords:** Chloroplast, *Rhododendron delavayi*, PacBio sequencing platform, phylogenetic analysis

## Abstract

*Rhododendron delavayi*, as a member of Ericaceae family, has been widely used as an important garden flower. The cp genome of *R. delavayi* exhibited a typical quadripartite cycle with 193,798 bp, comprising of a pair of inverted repeats (IRa and IRb) of 15,494 bp intersected by a large single copy (LSC) region of 160,234 bp and a quite small single copy region of 2576 bp. Totally, 123 unique genes were assembled in this cp genome, including 80 protein genes, 35 tRNAs and 8 rRNAs. Out of these assembled genes, 88 genes (71.54%) were single copy. Phylogenetic analysis based on 14 cp genome of related species showed that the *R. delavayi* was closely related to *Vaccinium oldhamii*. This study provides important information for future evolution, genetic and molecular biology studies of *Rhododendron*.

*Rhododendron delavayi* Franch belongs to Ericaceae family, a large and widespread woody plant genera comprising of over 1000 species throughout tropical southeast Asia (Ng and Corlett 2000). *R. delavayi* has bright and clustered flowers, long flowering period, and wide adaptability, which makes it a popular ornamental tree species in garden landscape. Due to lacking of molecular markers and genome information, the phylogenetic relationship of *R. delavayi* with other related species is still unclear. Recently, the chloroplast (cp) genomes have been widely used for phylogenetic reconstruction attributing to advances in high-throughout sequencing technology (Li et al. 2019; Yan et al. 2019). Herein, the complete cp genome sequence of *R. delavayi* was deciphered and the phylogenetic relationship with related species was also detected.

Total DNA was extracted from 5 g fresh leaves gathered from Dafang County, Guizhou Province, China (105°17′06′′E, 27°18′10′′N) using CTAB method stored in Guizhou Normal University (accession number: LJR-003). After DNA quality assessment, two DNA libraries with insert size of 20 kb and 400 bp fragments were constructed and then sequenced by PacBio Sequel platform and Illumina HiSeq 4000, respectively. The cp genome of *R. delavayi* was assembled based on cp-related reads by the Canu program using MHAP (MinHash Alignment Process) (Koren et al. 2017). For further correction and refinement, Then BLASR was used to map PacBio reads back to the assembly to refine the initial assembly. After that, Illumina reads were mapped to the assembly using BWA-MEM (Li 2014) with default parameters to further correct the assembly. The complete cp genome was submitted to GenBank (accession number of MN413198).

The complete cp genome sequence of *R. delavayi* is 193,798 bp in length, comprising of a large single-copy (LSC) region of 160,234 bp and a small single-copy (SSC) region of 2576 bp, which is separated by a pair of inverted repeat (IRa and IRb) regions of 15,494 bp. Totally, 123 genes were assembled, including 80 protein coding genes (12 genes with two copies), 35 tRNA genes (six genes with two copies and one genes with three copies) and 8 rRNA genes. Among these genes, 80 and 21 genes were distributed in LSC and IR regions, respectively, and only one gene located in SSC region. Overall GC content of the whole genome is 35.99%. And 303 simple sequence repeats loci with a length of at least 10 bp were identified in the whole cp genome by the web service of Misa.

Due to the lacing of cp genome information of Ericaceae family, 14 cp genome sequences from Ericales were downloaded from GenBank to construct phylogenetic trees by Neighbor-joining method of protein coding genes using MEGA7 (Kumar et al. 2016). The bootstrap analysis with 1000 replicates was used to confirm the stability of each tree node. Phylogenetic trees showed that *R. delavayi* was closely related to *Vaccinium oldhamii*. The complete cp genome sequence of *R. delavayi* can be great benefit to further study on its population genetics and molecular breeding (Figure 1).

**Figure 1.**
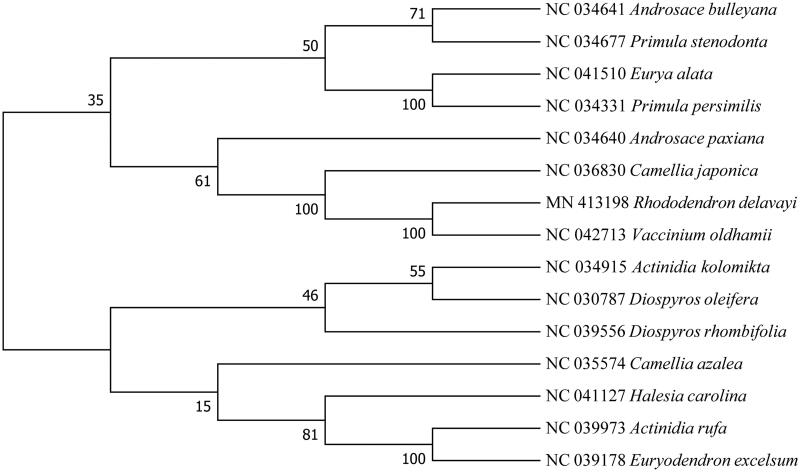
Phylogenetic trees of 15 Ericales species, based on protein-coding genes in the cp genome. The tree was generated by Neighbor-joining. The bootstrap consensus tree inferred from 1000 replicates is taken to represent the evolutionary history of the taxa analyzed.
